# Long-range movements coupled with heterogeneous incubation period sustain dog rabies at the national scale in Africa

**DOI:** 10.1371/journal.pntd.0008317

**Published:** 2020-05-26

**Authors:** Davide Colombi, Chiara Poletto, Emmanuel Nakouné, Hervé Bourhy, Vittoria Colizza

**Affiliations:** 1 INSERM, Sorbonne Université, Institut Pierre Louis d’Epidémiologie et de Santé Publique IPLESP, Paris, France; 2 Computational Epidemiology Laboratory, Institute for Scientific Interchange (ISI), Turin, Italy; 3 Physics Department and INFN, University of Turin, Turin, Italy; 4 Institut Pasteur de Bangui, Bangui, Central African Republic; 5 Institut Pasteur, Unit Lyssavirus Epidemiology and Neuropathology, WHO Collaborating Center for Reference and Research on Rabies, Paris, France; Universidad Nacional Mayor de San Marcos, PERU

## Abstract

Dog-transmitted rabies is responsible for more than 98% of human cases worldwide, remaining a persistent problem in developing countries. Mass vaccination targets predominantly major cities, often compromising disease control due to re-introductions. Previous work suggested that areas neighboring cities may behave as the source of these re-introductions. To evaluate this hypothesis, we introduce a spatially explicit metapopulation model for rabies diffusion in Central African Republic. Calibrated on epidemiological data for the capital city, Bangui, the model predicts that long-range movements are essential for continuous re-introductions of rabies-exposed dogs across settlements, eased by the large fluctuations of the incubation period. Bangui’s neighborhood, instead, would not be enough to self-sustain the epidemic, contrary to previous expectations. Our findings suggest that restricting long-range travels may be very efficient in limiting rabies persistence in a large and fragmented dog population. Our framework can be applied to other geographical contexts where dog rabies is endemic.

## Author summary

Rabid dogs constitute the main source of rabies transmission to humans, therefore understanding disease dynamics in dogs is essential to reduce human risk. Mathematical models are invaluable in exploring intervention scenarios where field experiments can be expensive or impractical. We developed a data-driven model to identify the mechanisms for rabies dispersal, accounting for dogs’ spatial fragmentation, human-mediated movements, and empirically estimated fluctuations of the incubation period in dogs. Applied to Central African Republic, our model indicates that continuous re-introductions of rabid dogs via human-mediated movements are critical in sustaining the disease in the country. Long-range travel restrictions can be very effective in aiding rabies control. Our approach can be extended to other geographical settings.

## Introduction

Canine rabies is a viral zoonosis responsible for approximately 21,500 (95% C.I. 9,100–58,100) annual human deaths in Africa [1]. Almost all cases result from bites from infected dogs, thus establishing domestic dogs as the primary vector for human rabies in the developing world [2,3]. Despite the existence of effective and efficient preventive measures [4–7], restricted availability [8,9], cost [8–10], cultural opposition [9,11] and lack of policy coordination hinder their full implementation in resource-limited countries [12–14]. As a result, rabies remains an endemic zoonosis throughout Africa, affecting individuals in urban areas as well as in remote or rural locations and continues to represent nowadays a major public health concern [2].

As rabid dogs constitute the main source of transmission to humans, understanding the dynamics of rabies in domestic dog populations is thus essential to identify the key drivers for pathogen maintenance and develop improved control measures to reduce spillover opportunities and, consequently, the number of human fatalities. An important indicator of the transmission potential of a disease is the basic reproductive number, *R*_0_, measuring the average number of secondary cases generated by a single infectious individual in a fully susceptible population [15,16]. Analyses of outbreak episodes based on surveillance of rabid dog cases reported rather low values of the transmission potential, with *R*_0_ estimates just above the critical value of 1, both in rural and urban areas in Africa [5,17–19]. Despite the low-level endemic stability that would require relatively low degrees of vaccination coverage to eliminate rabies [20], empirical and theoretical evidence suggests that sustained pulsed vaccination campaigns with 70% coverage of the domestic dog population are needed to achieve long-term elimination [17,21,22]. This high coverage is required to prevent the rapid decline of population herd immunity below its critical value between two consecutive campaigns, induced by the large renewal of the naive domestic dog population due to dogs’ short life-span and high fecundity rate [19,23].

However, the planning of intervention is made difficult by the geographical dissemination of the disease that may cause rabies reemergence in disease-free areas [19,23–25]. Rabies spatial dispersal is mainly ascribed to human-mediated transport of dogs [24,26–29]. Free-roaming movements of domestic dogs are indeed limited to very short distances of few kilometers per day [30], or less for rabid dogs [17]. Human-mediated movements were previously recognized to be an important vector for spatial dispersal of diseases in wildlife [31–33], domestic [26,27] and farmed animals [34]. For canine rabies, available evidence from household survey data indicates the rather common practice of transporting dogs with frequent movements (daily, monthly, and annual scales) even over large distances (e.g. between different provinces of a country) [29,35,36]. This may compromise vaccination campaigns unable to maintain high coverage over the entire region [24,29]. The key role played by humans in the dispersal of rabies is also evident from the observed relation between rabies virus spatial spread and the geographic location of human settlements, administrative borders, roads and bridges overcoming natural barriers [26,32,37–39].

Transmission of rabies occurs mainly through the bite of an infected animal, with inoculation of viral-loaded saliva in the subcutaneous or muscular tissue of a susceptible host [40,41]. The incubation period is highly variable, with a median close to one month, and a range that can extend from 10 days to more than one year [17]. This is likely related to the process of dissemination of the virus in the infected body, migrating from the site of inoculation to the central nervous system [42,43]. The infectious period is instead rather short, with a median around 3 days and fluctuations typically not exceeding 4 days. While dog movements are extremely restricted during the infective phase because of its duration and of disease symptoms, infected dogs may move across distant locations while incubating the virus, potentially carrying the disease over large distances, as suggested by phylogeographic data [26,27,35,39].

Several models were introduced to address the role of rabies reintroduction in disease-free areas through importations of rabies-exposed dogs [23,24,29,44–46]. Their findings are however specific to the characteristics of the public health and veterinary health interventions adopted (e.g. frequency of mass vaccination, coverage, movement bans, etc.). An endemic setting where no intervention has been introduced yet offers an ideal scenario to characterize the endemic disease dynamics at the spatial level, being it isolated from the effects resulting from disease control. It may thus provide the opportunity to better understand the underlying key mechanisms to guide the implementation of elimination measures. Central African Republic reported an endemic circulation of canine rabies for at least the last 20 years [18,47–49], with no mass vaccination campaign of domestic dog population implemented during the period. Long-term surveillance data on rabid-dog and exposed-human cases are available for the capital city of Bangui and showed that almost all exposed individuals (93%) are from Bangui or its immediate neighborhood in the suburban areas of Bimbo and Bégoua [47]. Analyses of epidemiological time series and viral sequence data indicated that the epidemiology of rabies virus in Bangui’s dog population is shaped by a sequence of successive waves characterized by periods of 53 and 89 months and long intervals (>1 year) of absence of cases [18]. This was associated to local extinction coupled with the introduction of new viral subtypes. Therefore, while canine rabies does not appear to self-sustain in the city of Bangui, its persistence was suggested to be related to frequent introductions of rabies-exposed dogs in the capital city from its neighboring area [18], a transmission pattern similar to those reported also in other African settings [17,19,25].

Here we investigate the factors underlying the observed rabies epidemiology in Bangui through a spatially explicit stochastic metapopulation model parameterized on the geography of Central African Republic and calibrated on 20 years epidemiological data from Bangui. We consider empirical distributions of incubation and infectious periods coupled with mapping of dog population in the country and models of human-mediated movements to study the local vs. long-range spatial transmission, accounting for both urban and rural settlements.

## Methods

### Metapopulation model structure

We develop a georeferenced discrete stochastic metapopulation epidemic model [50,51] for Central African Republic where domestic dog habitats are represented by model patches and human-mediated movements of dogs are represented by links connecting different patches. The infection dynamics occurs within each patch, and the disease can spread from one patch to another by means of dog movements.

Spatially distributed dog populations in the country are inferred from georeferenced high-resolution human demographic data [52–54], similarly to [45]. We used a publicly available high-resolution dataset of human population distribution in space [55] to map the georeferenced human settlements in the country and the associated population size, population density, and spatial extension. Population data is validated on Bangui population size provided by national census. The resulting human settlements in space correspond to the dog habitats considered in the metapopulation model, i.e. patches. They are classified as *urban* or *rural*, depending on the density of the corresponding human settlements. We follow the OECD (Organisation for Economic Co-operation and Development) definition using a threshold of 1,000 individuals/km^2^ to define a urban settlement [56]. Dog population size in each patch is computed from the human-to-dog ratio for *urban* (>1,000 individuals/km^2^, ratio = 21.20) or *rural* (<1,000 individuals/km^2^, ratio = 7.40) settings [53].

Human-mediated movements of dogs from one patch to another are modeled with a geographical distance model consistent with known patterns of human movements [57–60] and compatible with the spatial spread of canine rabies [35]. Similarly to [44], we define the number of dogs transported from one settlement to another to be proportional to 1/*d*, where *d* is the great circle distance between the centroids of the two patches. Movements are divided into three different ranges, similarly to [29]: *short-range* movements (*d*<20 km) correspond to the average distance within a municipality, *medium-range* movements (20≤*d*≤100 km) correspond to the average distance traveled to connect two municipalities within the same region, and *long-range* movements (*d*>100 km) correspond to the remaining ones.

### Infection dynamics and spatial spread

We model rabies virus transmission through mixing between individual hosts within the patches, and spatial dissemination through the explicit movements of discrete infected dogs between patches. Viral infection dynamics in dogs is described with a susceptible-exposed-infectious-removed (SEIR) compartmental model [15,16], as previously done for dog rabies [23,24,29,44–46,61], where hosts can be in one of the following states: susceptible (*S*), i.e. healthy individuals who may acquire the infection; exposed (*E*) hosts who have contracted the infection but do not shed the virus during an average incubation period *ε*^−1^; infectious (*I*) hosts who can transmit the virus for an average infectious period *μ*^−1^ following incubation; hosts removed (*R*) from the population as rabies is fatal once clinical symptoms appear [40]. More details are provided in the S1 Text. Accounting for the behavioral changes induced by the disease [17], rabid dogs are assumed to be restricted to the patch in which they are located at symptoms onset, whereas exposed dogs can migrate to different locations while incubating the virus according to the defined mobility model. The metapopulation model is parameterized with the empirical distributions for the duration of the incubation and infectious periods obtained for rabies virus in African domestic dog population [17]. To assess the role of the large fluctuations reported for the incubation period, we also consider another version of the compartmental model where disease progression occurs with constant rates, i.e. with exponentially distributed disease stages with the same average duration of incubation and infection, as done in [5,23,44,45]. We explore a range of values of the basic reproductive number (1.01≤*R*_0_≤1.16) consistent with previous estimates [5,17–19]. Vital dynamics is considered in the model to account for dogs’ high fecundity rate leading to a renewal of susceptibles and their relatively short average life span. Birth rate is found to vary drastically across different geographical areas [17,23,36,52,62–65], therefore we considered it as an unknown parameter of the model to be explored. Average life expectancy is parameterized with available estimates for domestic dogs in Africa (see S1 Text).

### Numerical simulations and analyses

We perform numerical simulations of rabies virus transmission in the modelled dog population. Simulations are discrete and stochastic to account for the discrete nature of hosts and for stochastic extinction events that may be favored by small host population sizes. Time is considered to be discrete with a daily timescale. Since the disease is endemic in the country [47], simulations are seeded in each patch with a proportion of exposed and infective dogs computed from surveillance data on rabid-dog cases in the capital city of Bangui [18], considering a detection rate of 20% as in [18]. Other initial conditions are explored in the S1 Text.

We considered different scenarios of transmission and birth rate. For each model parameterization and under each hypothesis considered, we run 10^3^ stochastic simulations for a long enough period (>300 years) to evaluate the occurrence of an endemic condition for rabies virus diffusion.

Simulations provide at each time step the number of domestic dogs in each compartment in each patch, and of those moving from one patch to another. We compute the persistence probability of rabies virus in the domestic dog population as the fraction of stochastic simulations reaching the endemic condition (i.e. pathogen survival in the dog population). Endemic prevalence per patch is computed as the average prevalence in time, excluding the initial transient period (~10 years), over each simulation run where rabies virus continued circulating in the dog population.

To estimate the reproductive number *R*_0_ and the birth rate, we fitted the metapopulation model to the epidemiological situation reported for Bangui. We used a Monte Carlo procedure to evaluate the likelihood, similar to what previously done in [66,67]. More details are reported in the S1 Text.

To estimate the degree of urbanization of rabies epidemic in Central African Republic compared to its dispersion in rural regions, we plot the national-level epidemic concentration curve (ECC) [68] by ranking patches according to increasing density *ϕ* of rabid dogs (*ϕ*_1_≤*ϕ*_2_≤⋯≤*ϕ*_*N*_) and computing the indicator $c_{j}=\frac{100}{\sum_{i}\phi_{i}}\sum_{i\geq j}\phi_{i}$.

The metapopulation framework is implemented in C++, and technical details for simulations are reported in the S1 Text. A sensitivity analysis is performed to assess the impact of numerical choices.

### Role of spatial fragmentation and human-mediated mobility

We numerically assess the impact of the landscape of human settlements in Central African Republic and of human-mediated movements on the persistence of canine rabies in the country by comparing our findings with synthetic scenarios altering the spatial structure of the dog population and/or its mobility, on the full parameter space explored. The scenarios considered are the following:

■ Only Bangui: Bangui is considered as an isolated patch, the rest of the metapopulation model is ignored;
■ Only Bangui neighborhood: transmission is considered exclusively in Bangui’s patch and its immediate neighborhood (<20km), assuming these patches are isolated from the other settlements;
■ Only urban patches: the metapopulation model is constituted exclusively of urban patches; rural patches and links departing from them are removed;
■ Only rural patches: the metapopulation model is constituted exclusively of rural patches; urban patches and links departing from them are removed.
■ Only short travels: all movements except short travels are restricted;
■ Only medium travels: as above for medium travels;
■ Only long travels: as above for long travels;
■ Only short + medium travels: long travels are restricted;
■ Only short + long travels: medium travels are restricted;
■ Only medium + long travels: short travels are restricted;

Additional scenarios (e.g. the whole country without the capital Bangui) are considered in the S1 Text.

## Results

### Domestic dogs’ spatial demographics and human-mediated movements

**Fig 1:**
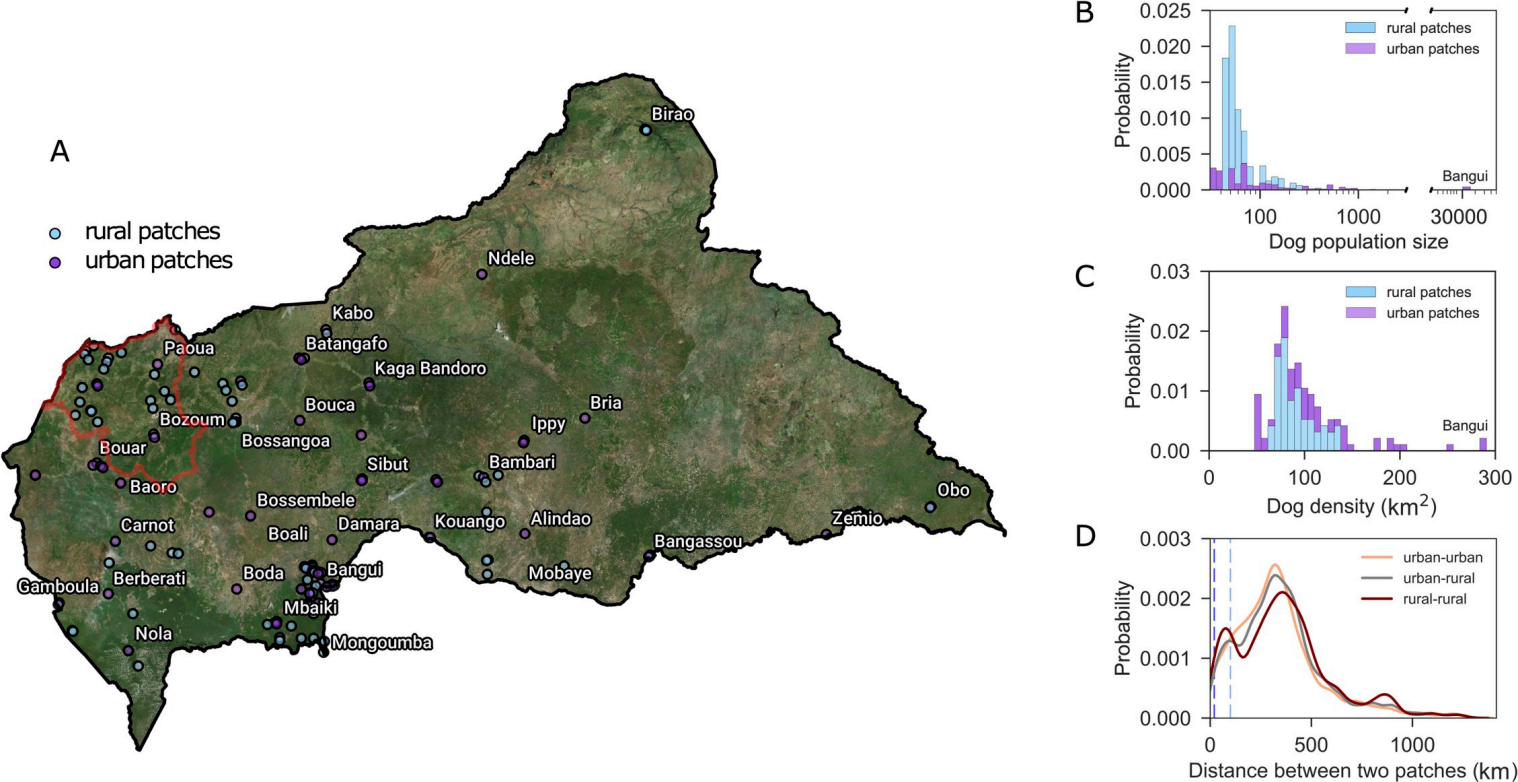
Modeled domestic dog population in Central African Republic. **A)** Geographical distribution of the predicted dog subpopulations divided in rural (light blue) and urban (violet) patches. The red shaped area indicates the Ouham-Pendé prefecture. **B)** Probability distribution of the estimated dog population size per patch in rural (light blue) and urban (violet) patches. **C)** Probability distribution of the estimated dog population density per km^2^ per patch in rural (light blue) and urban (violet) patches. **D)** Probability distribution of the distance between any two patches in the metapopulation network, whether connecting two urban patches (orange), two rural patches (dark red), a urban with a rural patch (black). The two dashed vertical lines indicate the separation between short, medium, and long-range movements. The satellite map was generated using data sourced from OpenStreetMap (OpenStreetMap contributors) and created through QGIS software (QGIS Development Team (2019). QGIS Geographic Information System. Open Source Geospatial Foundation Project. http://qgis.osgeo.org).

Dog population in Central African Republic is estimated to be of approximately 80,000 animals distributed in a rather dispersed spatial pattern comprising 137 settlements divided in 58 (42%) urban and 79 (58%) rural patches (Fig 1A). Only 10% of the total dog population lives in rural patches, whereas almost 50% is located in Bangui alone. Other top populated urban areas are Berbérati and Carnot in the western region of the country, accounting for 4% and 2.5% of the total dog population, and Bambari (3%) in the center-east.

Settlements are mainly gathered in the center and western regions of the country, with very few urban areas in the eastern region where national parks and natural reserves are located. Most rural settlements (>70%) have an estimated population size of less than 100 dogs, whereas the number of dogs in urban patches is rather heterogeneous, ranging from few tens up to the largest size constituted by the population in Bangui (almost 40,000 dogs, Fig 1B). Low populated urban patches are mostly geographically located in the proximity of the main cities. While dog population sizes change considerably between rural and urban settlements, their density per squared km are predicted to be rather similar, except for few urban patches with an estimated density of >150 dogs/km^2^ (Fig 1C).

**Fig 2:**
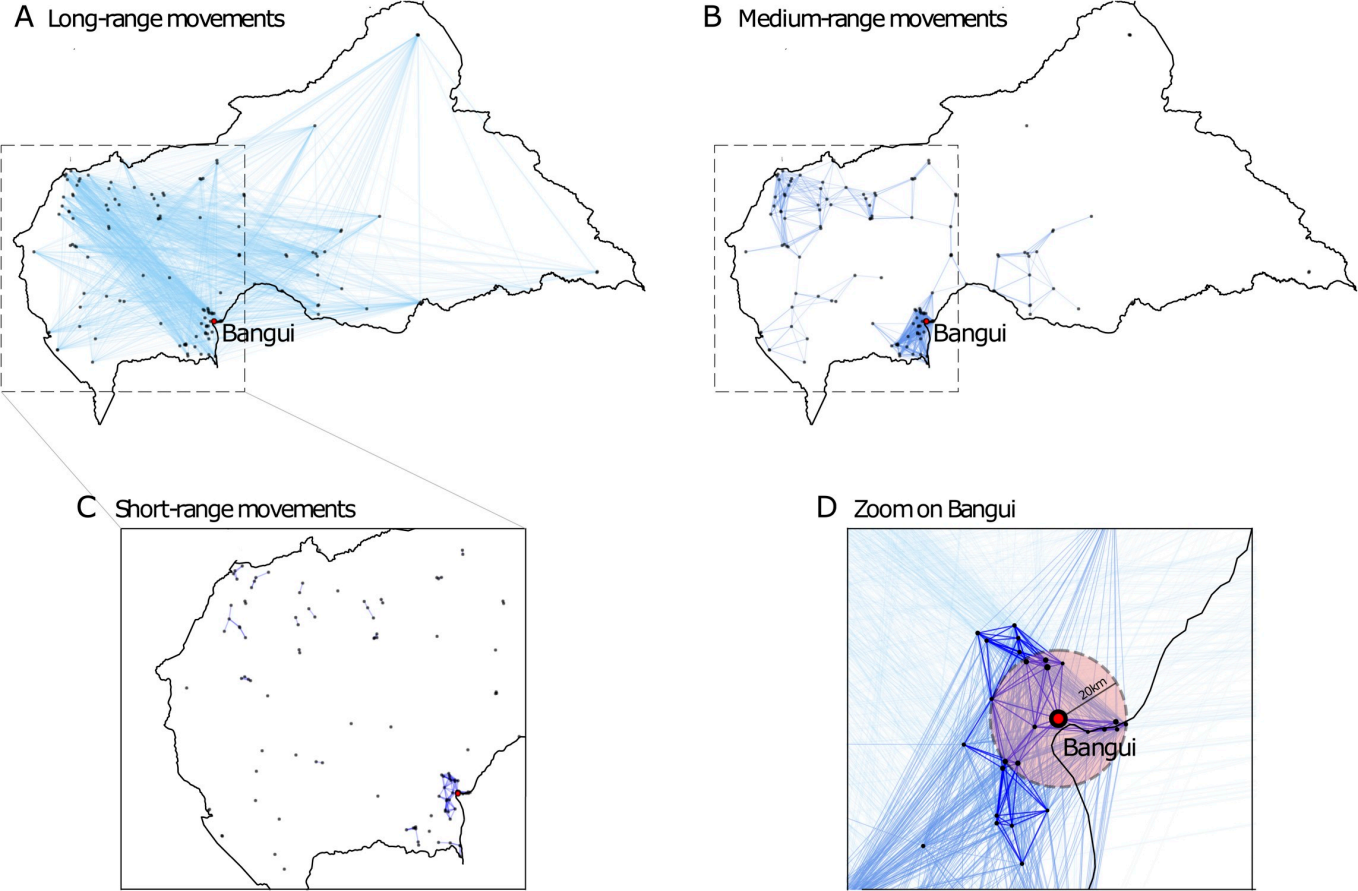
Georeferenced multiscale network of estimated domestic dog mobility. Dots represent patches and links represent the estimated human-mediated transports of dogs on long **A)**, medium **B)**, and short scales **C)**. **D)** Bangui and its surrounding region. The red circle defines Bangui’s neighborhood. The Central African Republic borders were generated using data from "Global Administrative Areas (2019)". University of California, Berkely. Available online: http://www.gadm.org [21/07/2019].

Given the country’s geography and population distribution, estimated human-mediated transport of dogs range from few kilometers for neighboring settlements to more than 1,000 km connecting far away locations (Fig 1D). The most probable distance traveled is predicted to be approximately 300 km for long-range movements connecting two urban patches or urban and rural patches, and it is slightly bigger (~350 km) for connections between two rural settlements. The latter type of movements displays in addition two marked peaks. The first is estimated at around 100 km and corresponds to a high spatial concentration of close rural habitats such as around the capital city or in the Ouham-Pendé prefecture in the northwest of the country (Fig 1A). The second is found for distances of approximately 850 km, typically connecting rural areas located in the northwest with the most populated areas in the center of the country (Fig 2A).

Multiple scales of connectivity are predicted by the mobility model for human-mediated movements of dogs (Fig 2). While long-range links are estimated to ensure the overall connectivity of the metapopulation, medium-range transports characterize mobility in the prefectures of Bangui and Ouham-Pendé in the north-west. Short-range transports move the largest number of dogs (estimated average of 0.035% of the dog population per day, Table 1) and provide connectivity to the local communities (40% of them are predicted to be located around Bangui).

**Table 1:** Modeled dog movements in Central African Republic. For each movement range we report the predicted number of links (and percentage of the total), and the predicted daily moving rate (dog/day).

Movement range	Estimated number of links (%)	Estimated % population moving daily
short (0–20 km)	183 (2%)	0.035 (95% CI: 0.003, 0.2)
medium (20–100 km)	951 (10%)	0.0046 (95% CI: 0.0001, 0.0146)
long (20 km–CAR borders)	8,182 (88%)	0.00064 (95% CI: 0.00002, 0.0025)

### Rabies virus persistence

**Fig 3:**
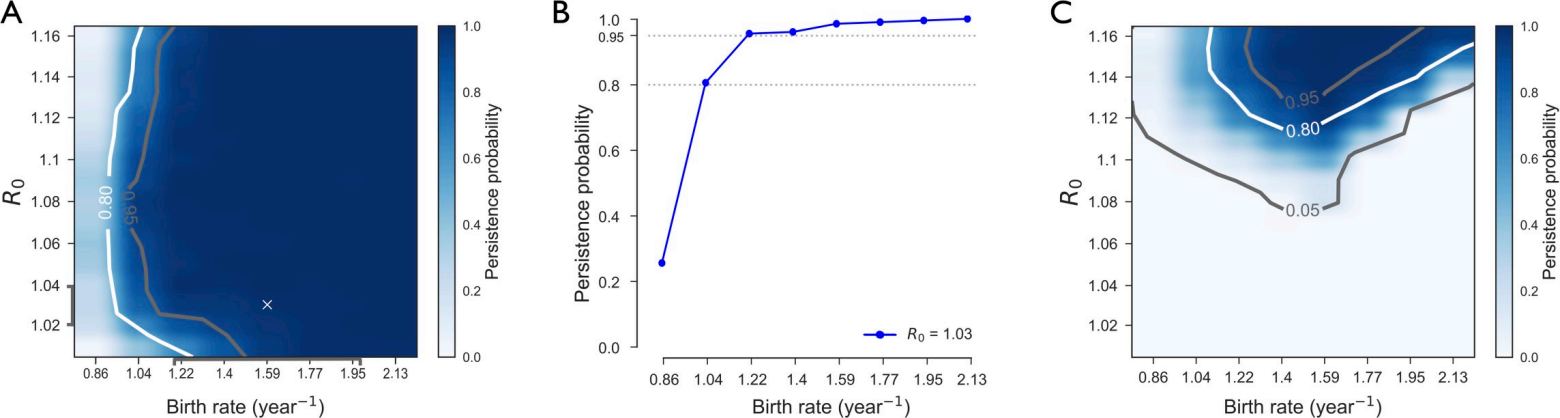
Dog rabies persistence probability in Central African Republic. **A)** Predicted persistence probability of rabies in the domestic dog population of Central African Republic as a function of the basic reproductive number *R*_0_ and of the annual birth rate. Results are obtained using empirically distributed incubation and infectious periods. The symbol ‘x’ corresponds to the maximum likelihood estimate. **B)** Predicted persistence probability as a function of the annual birth rate for the maximum likelihood estimate *R*_0_ = 1.03. The two dotted lines indicate 80% and 95% persistence probability. **C)** As in A) assuming that incubation and infectious periods are exponentially distributed with the same average duration of incubation and infection of the empirical distributions.

Rabies virus is predicted to remain endemic with high probability (≥80%) in Central African Republic for *R*_0_≥1.01 and a large range of birth rate values (Fig 3A); moreover, except for very low values of the basic reproductive number (*R*_0_≤1.02), persistence probability remains rather constant for increasing *R*_0_. Birth rates smaller than ≈1 dog/year lead to considerably rare maintenance of the virus, and a rapid transition toward high probability is predicted for increasing birth rates (Fig 3B). These findings are obtained parameterizing the infection dynamics with empirically estimated distributions of incubation and infectious periods. If instead we assume exponentially distributed disease stages, rabies virus would not be maintained in the domestic dog population, unless for higher values of the basic reproductive number (*R*_0_≥1.12, Fig 3C).

**Fig 4:**
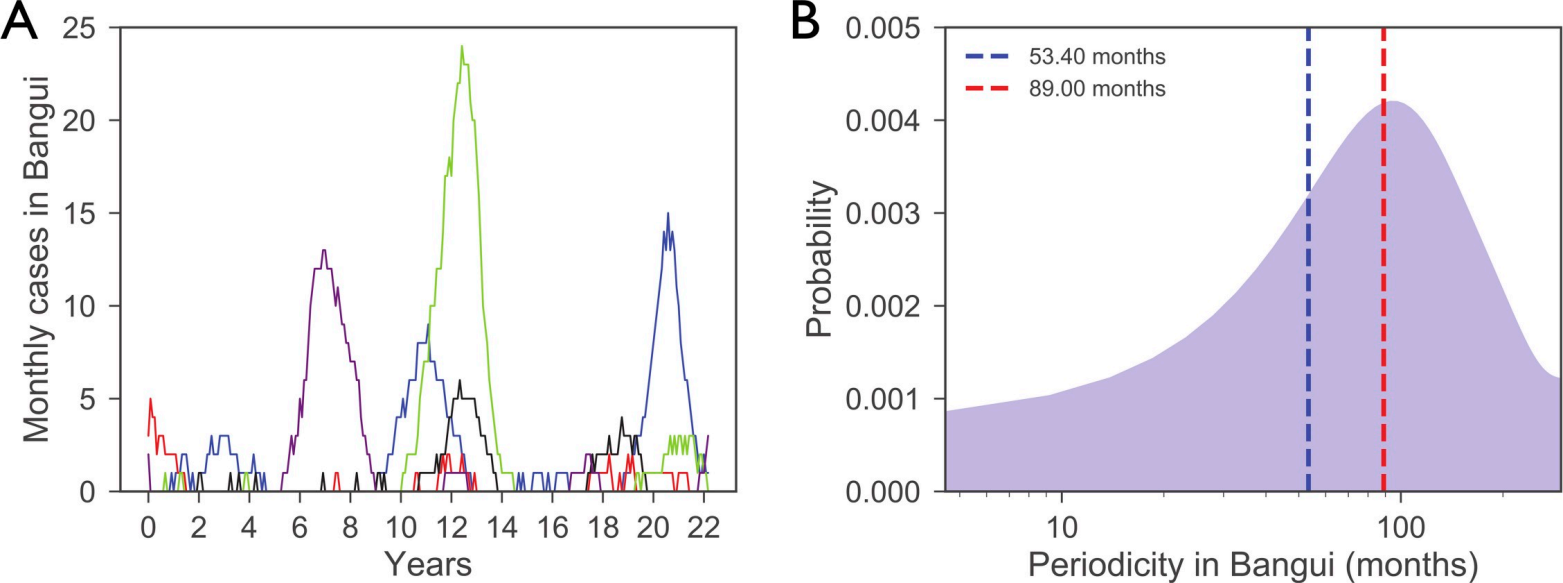
Numerical trajectories for Bangui. **A)** Simulated number of monthly cases in Bangui. Colors correspond to five different numerical runs. **B)** Probability distribution of the periods of the simulated epidemic cycles in Bangui compared to the two empirical oscillation periods of 53.4 (blue) and 89.0 months (red) reported in [18]. The distribution is obtained through wavelet analysis. Its peak is at 92 months. The x axis is in log scale.

We fitted parameters to the epidemiological surveillance data from Bangui estimating *R*_0_ equal to 1.03 (95% CI: 1.02–1.04) and birth rate equal to 1.59 (95% CI: 1.19–1.99). The monthly number of rabid dogs predicted in the city of Bangui ranges from zero to a few tens (Fig 4A), consistently with previous observations [18]. Wavelet analysis [69] was used to identify the periodicity of the numerical trajectories in Bangui and compare them with the estimates obtained from surveillance data in the city [18]. Numerically predicted epidemic waves in Bangui are characterized by a full spectrum of periodicities with the highest probability reached for the empirically estimated period of 89 months (Fig 4B). The distribution of the number of cases periodicity is obtained through wavelet analysis. Its peak is at 92 months. Additional numerical results are provided in the S1 Text.

### Spatial heterogeneity of rabies epidemic

**Fig 5:**
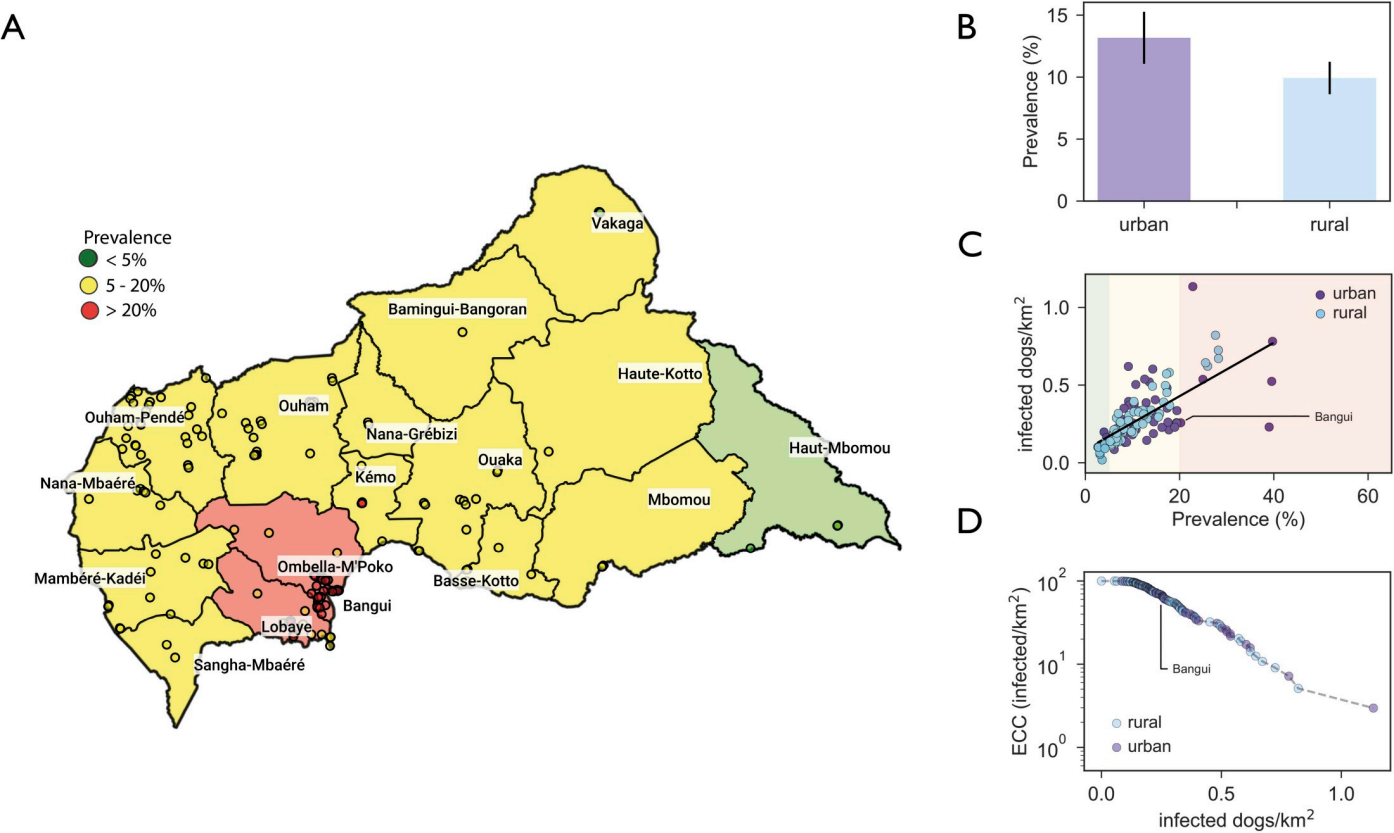
Dog rabies endemic prevalence in Central African Republic. **A)** Geographical representation of the predicted dog rabies endemic prevalence by administrative prefectures and by patch. Color codes range from low prevalence (<5%, green), to moderate prevalence (between 5% and 20%, yellow), to high prevalence (>20%, red). **B)** Predicted endemic prevalence by patch type. **C)** Scatter plot of the average density of infected dogs per km^2^ vs. the endemic patch prevalence. **D)** Epidemic concentration curve (ECC) for rabies prevalence in the domestic dog population in Central African Republic. ECC is used to address the relative contribution of high density and low density areas to endemic infection [68]. The Central African Republic borders and administrative areas were generated using data from "Global Administrative Areas (2019)". University of California, Berkeley. Available online: http://www.gadm.org [21/07/2019].

The map of Fig 5A provides a spatial visualization of the simulated endemic situation for canine rabies in the country. Most of the prefectures are predicted to have an average prevalence in the 5–20% range, with the exception of the most eastern prefecture of Haut-Mbomou at lower prevalence, and the prefectures of Bangui, Ombella-M’Poko and Lobaye at higher prevalence. These last prefectures also contain the settlement with the highest proportion of rabid dogs, in both rural and urban patches. Prevalence is predicted to vary depending on the settlement type (Kolmogorov-Smirnov test, *p* = 0.0001, Fig 5B) and correlates with the density of infected dogs in each patch (Pearson correlation *r* = 0.7,*p*<10^−6^, Fig 5C).

Fig 5D shows the lack of marked degree of urbanization of rabies epidemic in the country, with both urban and rural patches continuously contributing to both prevalence and density of infections.

### Role of spatial fragmentation and human-mediated mobility

**Fig 6:**
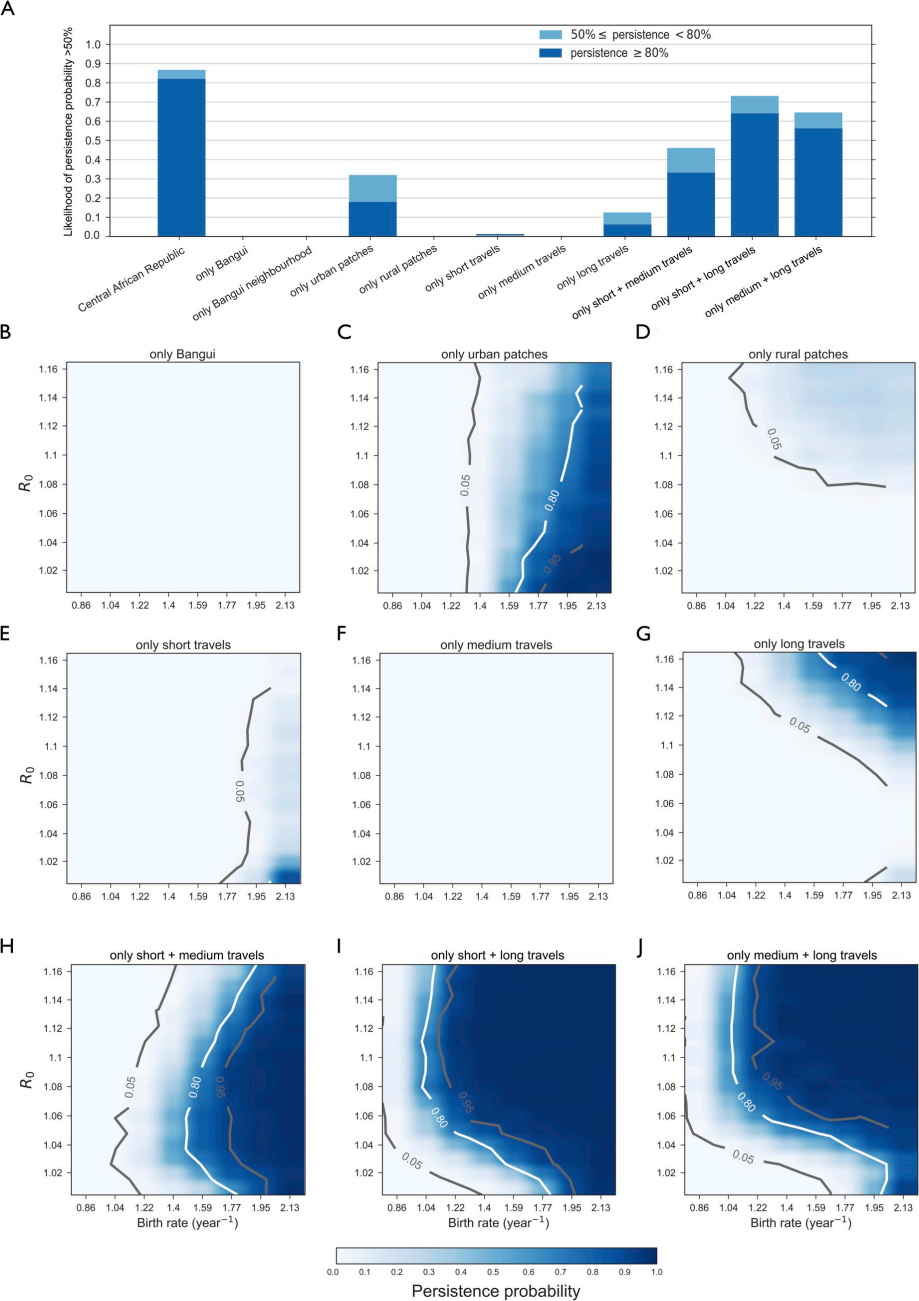
Role of spatial fragmentation and mobility. **A)** Likelihood to have persistence probability between 50% and 80%, or larger than 80% (light blue/dark blue stacked histogram) in the full parameter space (*R*_0_, birth rate) investigated and for different experimental scenarios (panels B-J). **B), C), D), E), F), G), H), I), J)** Predicted persistence probability of rabies in the domestic dog population as a function of the basic reproductive number *R*_0_ and of the annual dogs birth rate for the scenarios considering: only Bangui population B); only urban patches and their connections, discarding rural patches C); only rural patches and their connections, discarding urban patches D); all patches but connected only through short travels E), medium travels F), or long travels G); all patches but connected only through short and medium travels H), short and long travels I), or medium and long travels J).

Transmission in Bangui only, or in the network of Bangui and its neighborhood would not self-sustain (Fig 6, panels a and b, and Fig 2 in S1 Text). Focusing on the role of urban vs. rural areas, our model predicts that rabies would not be able to circulate in a set of connected rural patches only (panels a and d), whereas its persistence probability would increase if dog population were structured in urban patches only (Fig 6C). Removing Bangui leads to results similar to urban patches only scenario (Fig 6 in S1 Text).

Restrictions of human-mediated movements is predicted to dramatically impact viral persistence at the country level. Considering one single range of movement–whether short, or medium, or long–would practically prevent the virus to persist (Fig 6E, 6F and 6G), even if the mobility network still ensures connectivity between the country’s settlements (see Table 2 in S1 Text). Coupling two scales of movements in the model would re-establish similar persistence probability profiles as in the full model if long-range movements are present (Fig 6I and 6J). If dogs were not allowed to move on such long-range connections, the probability for a considerably high persistence would decrease substantially (Fig 6H).

## Discussion

Controlling and eliminating rabies in domestic dogs through the implementation of strict control measures including dog mass vaccination campaigns is now considered as the most cost-effective measure to prevent rabies in humans [6,13,17,24]. Thousands of people however continue to die every year from rabies disease transmitted by infected dogs, and rabies remains endemic throughout the African continent. In most African countries rabies surveillance is possible only in capital cities, where external re-introductions of the disease are suggested to sustain persistence [18,19,70]. On the other hand, very little is generally known about how the transmission in rural and periurban areas can affect the epidemic in the cities. Understanding the key drivers for the endemic circulation of the virus on a large geographical area is thus essential to design improved interventions for a sustainable elimination of dog rabies. Focusing on Central African Republic where the virus has been endemic in the domestic dog population for more than 20 years [18,47], we introduce a spatially explicit model to simulate rabies transmission within both villages and urban areas and the spatial diffusion of the epidemic throughout the country led by human-mediated movements. Through numerical simulations, we can investigate the role of the various components acting on the infection dynamics at the population level.

First, we find that rabies would go extinct not only in the capital city alone, but also considering continuous re-introductions from the neighborhood of the city. This is in sharp contrast with previous expectations of the pattern of viral circulation proposed for Bangui [18] and other capital cities, such as e.g. N’Djamena in Chad [5,19], which suggested that importations might occur from the peri-urban areas adjacent to the city [18,19]. Our model predicts that longer range movements, connecting far-away settlements, are essential for rabies persistence. They allow occasional reintroductions of the disease sustaining its spatial spread.

Further, our work clearly shows that persistence of rabies in Central African Republic is favored by the non-trivial interplay of long-range movements of infected dogs with unusually long incubation periods. Thus, the virus can slowly diffuse in the country and the resulting continuous seeding of asynchronous rabies waves in distant settlements can efficiently counteract the local viral fade out while susceptibles are swiftly renewed elsewhere. This effect was already found in theoretical studies and in prior work addressing other diseases [71–75]. It was only recently considered for canine rabies persistence [44,45]. Moreover, this effect may be relevant for rabies in other carnivore species behaving in a similar way. It is indeed now evident that rabies virus has long evolved mechanisms to avoid extinction and successfully maintain itself in different reservoir host species during several thousand years, often in fragmented and poorly connected populations [76,77]. The intensification and the change of scale of human-mediated dissemination starting in the 15th century only plaid a role in the rapid global spread and maintenance of rabies observed in the 18th and 19th century in the Americas, Africa, Asia and Europe [76]. The prolonged incubation periods occurring in nature in dogs could then be an intrinsic factor of rabies virus pathogenesis that have constantly been selected and maintained during rabies virus genetic evolution as it played an instrumental role to ensure viral dispersion to new dog populations allowing the renewal of naïve domestic dog populations.

Our model also allows to address the respective role of urban and rural areas and shows that both are important for disease persistence, but extinction is more likely in a network of connected rural patches (as if, for example, urban areas undergo massive vaccination). This seems to be unrelated to the density of dogs–as it is rather similar in urban and rural patches with few exceptions, leading to a predicted spatial epidemic not showing a marked degree of urbanization–and it is more likely associated with the overall larger dog population size localized in urban patches.

Finally, numerically obtained periodic patterns of infected dogs in time are compatible with observations in Bangui [18] and in other African countries [17,25]. While we find a continuum spectrum of possible period durations that were not captured by surveillance data, this is likely due to the size effects of the limited observations (20 years) compared to the numerical extension of our simulations (>300 years). Analysis of longer historical data, when available, can further validate our predictions.

Our modeling findings have significant implications for the control of canine rabies in Central African Republic. The predictions indicate that sustainable elimination of the disease will indeed require a substantial effort targeting a much larger geographical area than focusing exclusively on the capital city and its surrounding peri-urban settlements. To be effective, mass vaccination campaigns would require targeting the entire set of urban areas in the country (42% of the settlements), which may result to be unfeasible. Banning movements on the long range (associated to the smallest fluxes of dogs moved) may be very effective in supporting sustained and prolonged vaccination campaigns in the country.

Our estimates indicate a low-level endemic stability of the disease consistent with previous values obtained in Bangui and in other African countries, and allowing rather steady dog population sizes [17–19]. With no mass vaccination in Central African Republic, higher transmission scenarios and the fatality rate of the disease would indeed not be compatible with the observed rather stable host population [24,78,79].

Our study is affected by some limitations. First, model inputs of host population size and mobility are inferred from human data and mobility models, similarly to [44,45]. Lack of data characterizing hosts’ spatial distribution and mobility is a central issue for epidemic models, especially in African countries, where also data on human movements are typically scarce [80]. For this reason, well-established models of human mobility, such as e.g. the gravity model [81], are largely used in spatially explicit epidemic models for human diseases. Here, we use the same approach to model human-mediated movements of dogs from one settlement to another. Additional refinements to the gravity model can be made in future work (e.g. a refined dependence on the distance between settlements), if surveillance data from multiple locations (and not only from the capital city) become available. Although a direct comparison is limited by the differences in contexts, we find that our parsimonious choice of the gravity model provide projections for canine population moving daily on short, medium, and long-range movements that are close to the upper estimates resulting from a 2013 survey on human-mediated dog movements in the Philippines [29]. While lack of data remains a limitation, our modeling approach offers the theoretical framework where to evaluate the role that spatial fragmentation of human settlements in a particular geographical area may have on rabies persistence, based on expected human mobility flows. As such, it can be readily applied to other countries and different landscapes. This may additionally provide numerical evidence for context-specific elements favoring rabies dispersal (e.g. geography, natural barriers, etc.). Second, we did not consider rabies virus transmission from wildlife to domestic dogs, as dogs are recognized to be the main reservoir for rabies in sub-saharan Africa [32,63,64] and dog-to-dog transmission is estimated to be approximately eight times as common as transmission between dogs and other carnivores [30,63,82]. Moreover, domestic dogs live mostly in human settlements where interactions with wildlife are sporadic. Indeed epidemiological cycles of the virus in non-flying wildlife mammals are geographically limited in Africa and, to our knowledge, not present in Central African Republic [76]. Third, like other studies [83], our model did not consider the role of density transmission of RABV and assume a homogeneously mixing dog population within each patch. Therefore, it neither accounts for the fine scale heterogeneities in dog densities nor for the network structure of dog-to-dog contacts within the patches [84]. The study of the role of contact patterns between dogs is still at its infancy, and additional empirical data are needed to infer synthetic models that can be applicable in space. Fourth, we did not consider possible importation events across the country borders. This may occur for instance in the north along the border with Chad, separating densely populated areas in both countries. Future work may further extend our framework to a larger region including several countries where rabies disease is endemic in the domestic dog population. Increasing evidence calls indeed for prolonged and sustained efforts that are concerted across different key stakeholders and countries in the region [4,25,85,86]. By parameterizing the model to national contexts, including the spatial fragmentation of the host population and implemented interventions, and fitting it to available surveillance data, it would be possible to numerically investigate the impact of different degrees of cross-border mobility on rabies persistence, and provide novel fundamental understanding to guide the successful elimination of rabies in the region.

## Supporting information

S1 Text The file contains: mathematical formulation of the models, additional numerical results and input values for the models. (DOCX)
