# Supplementary material for: Long-range movements coupled with heterogeneous incubation period sustain dog rabies at the national scale in Africa
Source: PLoS Negl Trop Dis. 2020 May 26;14(5):e0008317. doi: 10.1371/journal.pntd.0008317 (PMC7274467; doi:10.1371/journal.pntd.0008317)
Supplement: S1 Text — (DOCX) [file pntd.0008317.s001.docx]

**S1 Text**

**Long-range movements coupled with heterogeneous incubation period sustain dog rabies at the national scale in Africa**

Davide Colombi^1,2,3^, Chiara Poletto^1^, Emmanuel Nakouné^4^, Hervé Bourhy^5^, Vittoria Colizza^1*^

1) INSERM, Sorbonne Université, Institut Pierre Louis d’Epidémiologie et de Santé Publique IPLESP, F75012, Paris, France

2) Computational Epidemiology Laboratory, Institute for Scientific Interchange (ISI), Via Chisola 5, 10126 Turin, Italy

3) Physics Department and INFN, University of Turin, via P. Giuria 1, 10125 Turin, Italy

4) Institut Pasteur de Bangui, BP 923, Bangui, Central African Republic

5) Institut Pasteur, Unit Lyssavirus Epidemiology and Neuropathology, WHO Collaborating Center for Reference and Research on Rabies, 25-28 Rue du Dr Roux, 75015 Paris, France

*Corresponding author

Vittoria Colizza

INSERM, Sorbonne Université

Institut Pierre Louis d’Epidémiologie et de Santé Publique (IPLESP)

27 rue Chaligny

75012 Paris, France

Email: vittoria.colizza@inserm.fr

Phone number: +33 1 44 73 84 59

Fax: +33 1 44 73 84 62

Table of content

[1. Mathematical formulation of the model 3](#_Toc30077374)

[1.1 Rabies transmission dynamics with empirical distributions for incubation and infectious periods 3](#_Toc30077375)

[1.2 Rabies transmission dynamics with exponential distributions for incubation and infectious periods 7](#_Toc30077376)

[2. Inferring dog population from human population data 7](#_Toc30077377)

[3. Simulation details 9](#_Toc30077378)

[3.1 Initial conditions 9](#_Toc30077379)

[3.2 Stochastic and discrete integration of the disease dynamics 9](#_Toc30077380)

[3.3 Stochastic dog movements 11](#_Toc30077381)

[4. Maximum likelihood estimation 12](#_Toc30077382)

[5. Details of the experimental scenarios 13](#_Toc30077383)

[6. Sensitivity analysis 16](#_Toc30077384)

[6.1 Carrying capacity 16](#_Toc30077385)

[6.2 Sensitivity of surveillance system and initial conditions 17](#_Toc30077386)

[7. Additional numerical results 18](#_Toc30077387)

## Mathematical formulation of the model

- 1. Rabies transmission dynamics with empirical distributions for incubation and infectious periods

The mathematical formulation of rabies virus transmission dynamics follows a SEIR compartmentalization: susceptible $\left( S \right)$ hosts can be infected becoming exposed $\left( E \right)$; after the incubation infected dogs become infectious ($I$) and finally die ($R$). To incorporate the empirical gamma-distributed incubation and infectious periods in the compartmental model, we used the approach already adopted by [1,2]. In this approach both infected compartment are divided into *m* and *n* subclasses that corresponds to the shape of the gamma distribution of the incubation and infectious periods respectively:

$f_{m}\left( \tau_{E};\sigma\right)=\frac{\tau_{E}^{m-1}e^{{-\tau_{E}}/\sigma}}{\sigma^{m}\Gamma\left( m \right)}$ and $f_{n}\left( \tau_{I};\alpha\right)=\frac{\tau_{I}^{n-1}e^{{-\tau_{I}}/\alpha}}{\alpha^{n}\Gamma\left( n \right)}$

where $m$, $n$ are the shapes and $\sigma$ , $\alpha$ are the scale parameters of the exposed and infectious distributions. The average incubation and infectious periods are respectively $\varepsilon^{-1}=\sigma m$ and $\mu^{-1}=\alpha n$ (Table 1). Therefore, in the SEIR model, the transitions are described as:

$$\begin{matrix} E\to I \text{with rates }\frac{f_{m}\left( \tau_{E}\Delta t \right)}{1-F_{m}\left( \tau_{E} \right)} \end{matrix}$$

$$I\to R \text{with rates }\frac{f_{n}\left( \tau_{I}\Delta t \right)}{1-F_{n}\left( \tau_{I} \right)}$$

$F_{m}$ and $F_{n}$ are respectively the two distribution functions of $f_{m}$ and $f_{n}$. We consider in the following deterministic continuous differential equations for the sake of simplicity in explaining the model, whereas simulations are discrete and stochastic (see Subsection 3.2). The index *i* indicates the patch *i* of the metapopulation network. Disease progression is mathematically described by the following equations:

$$\frac{dS_{i}}{dt}=bN_{i}-\beta\frac{S_{i}I_{i}}{N_{i}}-{\kappa_{i}S}_{i}$$

$$\frac{dE_{1,i}}{dt}=\beta\frac{S_{i}I_{i}}{N_{i}}-\left( \sigma^{-1}+\kappa_{i} \right)E_{1,i}$$

⁞

$$\frac{dE_{m,i}}{dt}=\sigma^{-1}E_{m-1,i}-\left( \sigma^{-1}+\kappa_{i} \right)E_{m,i}$$

$$\frac{dI_{1,i}}{dt}=\sigma^{-1}E_{m,i}-\left( \alpha^{-1}+\kappa_{i} \right)I_{1,i}$$

⁞

$$\frac{dI_{n,i}}{dt}=\alpha^{-1}I_{n-1,i}-\left( \alpha^{-1}+\kappa_{i} \right)I_{n,i}$$

$$\frac{dR_{n,i}}{dt}=-\alpha^{-1}I_{n,i}$$

$$\frac{dN_{i}}{dt}=\left( b-d \right)\left( 1-\frac{N_{i}}{K_{i}} \right)N_{i}-\mu I_{i}$$

where $\kappa_{i}=d+\left( b-d \right)\frac{N_{i}}{K_{i}}$ is the density dependent death rate, $N_{i}$ is the total population and $K_{i}$ is the carrying capacity of patch *i* (see Table 2). This variable, in ecology, corresponds to the maximum population size of a certain species that the environment can sustain indefinitely, given the food, habitat, water, etc. We set it at the patch level and different values are explored, as it is the resulting outcome of the interplay of multiple non-trivial factors that are very challenging to estimate. The intrinsic $R_{0}$value, the basic reproductive number in the absence of migration per each patch *i*, is obtained using the next generation matrix approach [3], considering the multiple stages as in [1,2]:

$R_{0}=\frac{\beta}{\alpha^{-1}+\kappa}\left( \frac{\sigma^{-1}}{\sigma^{-1}+\kappa} \right)^{m}\cdot\sum_{j=0}^{n-1} \left( \frac{\alpha^{-1}}{\alpha^{-1}+\kappa} \right)^{j}$ (1)

The force of infection in a given patch *i* at time *t* is:

$\lambda\left( t \right)=R_{0}\frac{\alpha^{-1}+\kappa}{\left( \frac{\sigma^{-1}}{\sigma^{-1}+\kappa} \right)^{m}\cdot\sum_{j=0}^{n-1} \left( \frac{\alpha^{-1}}{\alpha^{-1}+\kappa} \right)^{j}}\frac{I_{i}\left( t \right)}{N_{i}\left( t \right)}$ (2)

where $I\left( t \right)$ and $N\left( t \right)$ are the number of infectious individuals and the total size of the population at time $t$, $R_{0}$ is the basic reproductive number.

In our numerical simulations, we fix a value for $R_{0}$ (and then explore a full range of values) and compute the corresponding forces of infection.

**Table 1.** **Parameters description and values**. We provide here a table listing the model parameters and their values.

| Notation | Parameter description | Value |
| --- | --- | --- |
| $\varepsilon^{-1}$ | average incubation period | 22.11 days [4] |
| $\mu^{-1}$ | average infectious period | 3.1 days [4] |
| $d^{-1}$ | life span | 2.2 years [4] |
| $m$ | shape of gamma distribution(incubation period) | 2.0 [4] |
| $\sigma$ | scale parameter of gamma distribution (incubation period) | 11.055 [4] |
| $n$ | shape of gamma distribution(infectious period) | 3.0 [4] |
| $\alpha$ | scale parameter of gamma distribution (infectious period) | 1.1 [4] |
| $K_{i}$ | carrying capacity | $N_{i}$, ${5N}_{i}$ (Bangui)  3$N_{i}$ [2-5$N_{i}$] (Central African Republic) |
| $R_{0}$ | basic reproductive number | 1.01-1.17 (Central African Republic)[4]  1.01 – 2.0 (Bangui) |
| $b$ | annual birth rate | 0.86 - 2.13 dogs per year [4,5] |
| $\rho$ | detection probability | 20% [5%, 10%, 50%]­ [6] |

- 1. Rabies transmission dynamics with exponential distributions for incubation and infectious periods

In the following, we show the mathematical description of the disease progression that we used for the compartmental model with exponentially distributed disease stages:

$$\frac{dS_{i}}{dt}=bN_{i}-\beta\frac{S_{i}I_{i}}{N_{i}}-{\kappa_{i}S}_{i}$$

$$\frac{dE_{i}}{dt}=\beta\frac{S_{i}I_{i}}{N_{i}}-\left( \varepsilon+\kappa_{i} \right)E_{i}$$

$$\frac{dI_{i}}{dt}=\varepsilon E_{i}-\left( \mu+\kappa_{i} \right)I_{i}$$

$$\frac{dN_{i}}{dt}=\left( b-d \right)\left( 1-\frac{N_{i}}{K_{i}} \right)N_{i}-\mu I_{i}$$

The intrinsic $R_{0}$ value obtained with the next generation matrix approach [3] is given by:

$R_{0}=\frac{\beta\sigma}{\left( \varepsilon+\kappa_{i} \right)\left( \mu+\kappa_{i} \right)}$ (3)

The force of infection in a given patch *i* at time *t* is:

$\lambda_{i}\left( t \right)=R_{0}\frac{\left( \varepsilon+\kappa_{i} \right)\left( \mu+\kappa_{i} \right)}{\beta\sigma}\frac{I_{i}\left( t \right)}{N_{i}\left( t \right)}$ (4)

## Inferring dog population from human population data

Domestic dogs are naturally tied to human population therefore we infer dog densities starting from human demographics and human geography [7,8]. To capture the highly fragmented and complex landscape given by the distribution of dogs communities in Central African Republic we employed as a proxy a high resolution dataset on human population called *WordPop* [9].

**
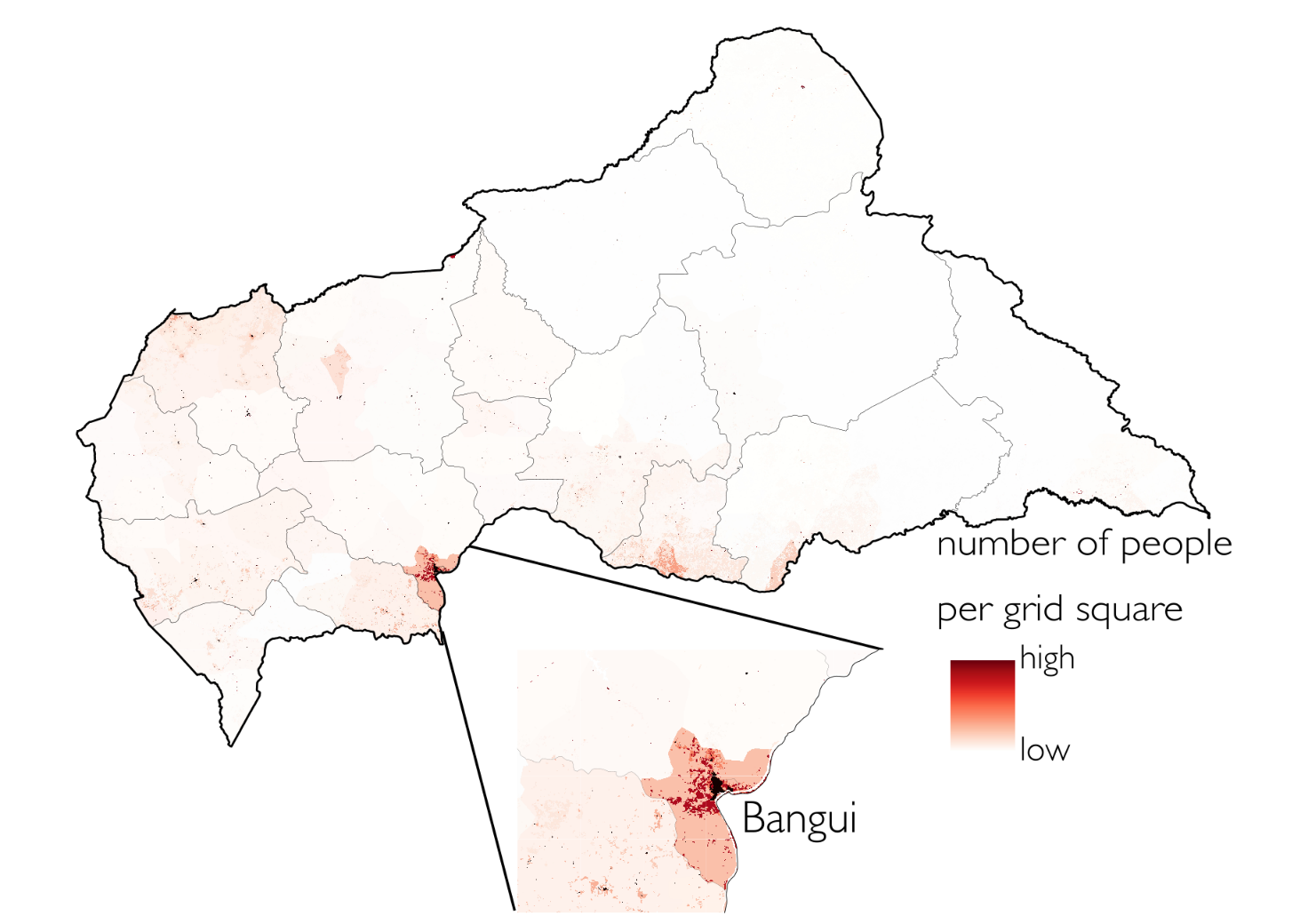
**

**Fig 1. WorldPop Central African Republic.** 2015 estimates of numbers of people per grid square of Central African Republic, with national totals adjusted to match UN population division estimates. Resolution of 0.000833333 decimal degrees (approx 100m at the equator).

WorldPop contains estimates for the geographic distribution of the population in terms of population density and settlement patterns for 126 countries in Africa, America, and Asia. Fifty African countries, including Central African Republic, are represented in the database. For our analyses, we used the alpha version of the WorldPop data set for Central African Republic, containing 2015 estimates of the number of people/100 m^2^. The dataset is a raster image, and it is composed of a set of discrete uniform cells on a gridded surface (pixels) associated to an estimation of the human population. The WorldPop database was constructed by using satellite data on surface imagery, specifically imagery on land cover patterns, to map the settlement patterns. The surface imagery data were used to reallocate the population census data to settlements; settlements may vary from cities to small rural homesteads. The details of the methodologies used by *Linard et al.* [10,11] to construct the database are described on the WorldPop website.

The Central African Republic dataset comprises 165,076,538 cells of which 72,939,427 are populated. To design the metapopulation model we aggregate data from the raster dataset to satisfy two conditions:

- neighbouring inhabited cells (i.e. sharing one border) are merged together in the same settlement;
- Bangui, the capital city of the country, is recovered as a single patch in the metapopulation network with a population close to current census estimates (734,350 inhabitants in 2012)

We adjusted the raster dataset resolution (exploring from 100 m^2^ to 1 km^2^) and filtered out scarcely populated cells (exploring threshold values from 20 to 60 individuals/ km^2^) to fulfil these conditions, and obtained a georeferenced metapopulation network of 137 human settlements. From this, we inferred the metapopulation of spatially disperse communities of domestic dogs, as illustrated in the main paper.

## Simulation details

- 1. Initial conditions

Since the disease is endemic in the country [6,12] each patch $i$ is seeded with a certain fraction of exposed and infectious individuals. The fraction of infected individuals is obtained from the estimate resulting from Bangui’s surveillance data, and assuming the detection probabilities assumed in [6]. The proportion of exposed in endemic condition is then obtained through simple numerical calculations. Varying initial conditions are considered for sensitivity analysis.

- 1. Stochastic and discrete integration of the disease dynamics

In each patch *i* for a compartment $X_{i}$we extract a random variable from a multinomial distribution for each possible transition out of the compartment in the discrete time interval $\Delta t$. Such variable, $D_{i}\left( X_{i},Y_{i} \right),$determines the number of transitions from the compartment $X_{i}$ to a given compartment $Y_{i}$ occurring in $\Delta t$. The change of population size of a compartment in the time interval because of the disease dynamics is given by the sum over all random variables extracted, each for a specific transition:

$\Delta X_{i}=\sum_{Y} \left[ -D_{i}\left( X_{i},Y_{i} \right)+D_{i}\left( Y_{i}{, X}_{i} \right) \right]$ (5)

Here we consider the evolution of the susceptible compartment $S_{i}$ as a concrete example. All possible transitions from this compartment are: to the exposed $E_{i}$; to the natural death given by the demography.

The random variables for these transitions from $S_{i}$ are extracted from the multinomial distribution:

${Pr}^{multin}\left( S_{i},P_{S_{i}\to E_{i}},{-P}_{S_{i}\to death} \right)$ (6)

with the transition probabilities:

- $P_{S_{i}\to E_{i}}=\lambda\Delta t$
- $P_{S_{i}\to death}=d\Delta t$

These two transitions cause a reduction of the size of that compartment $S_{i}$. The increase is given by the birth of the domestic dogs. Also in this case the transition is modeled by a random number extracted from a binomial distribution:

${Pr}^{bin}\left( S_{i},P_{birth\to S_{i}} \right)$ (7)

with probability $P_{birth\to S_{i}}=b\Delta t$*.* After extracting these numbers from the corresponding distributions, we can calculate the stochastic variation of the population size of compartment $S_{i}$:

$\Delta S_{i}\left( t \right)=S_{i}\left( t+1 \right)-S_{i}\left( t \right)=-\left[ D_{i}\left( S_{i},E_{i} \right)+D_{i}\left( S_{i},death \right) \right]+D_{i}\left( birth,S_{i} \right)$ (8)

$\Delta t$ is equal to 1 day.

- 1. Stochastic dog movements

The number of dogs that migrate from patch $i$ to patch $j$ is given by [13]:

$$w_{ij}=\frac{C}{d_{ij}}\Delta t$$

with $\Delta t$ = 1 day. $C$ is a normalization factor that fixes the maximum number of dogs that can daily escape from each subpopulation to 1% of the patch population. This threshold allows to have a percentage of the canine population that moves daily that is in agreement with the estimate of human-mediated dog movements made in the Philippines [14].

Considering patch $i$, with $n$ that indicates all the other patches connected to $i$, the computation of $C$ is the following:

$\sum_{j}^{n} w_{ij}\leq0.01 N_{i}$

$\sum_{j}^{n} \frac{c_{ij}}{d_{ij}}\Delta t= \frac{C_{i}}{d_{i}^{tot}}\leq0.01 N_{i}$

$$C_{i}\leq0.01 N_{i} \cdot d_{i}^{tot}$$

For each patch $i$. In order to have a single parameter for the whole metapopulation network we computed $C$ as:

$C=0.01\cdot\min_{i=0, ..,n} (\left\{ N_{i} \cdot d_{i}^{tot} \right\})$

The number dogs in the compartment $X_{i}$ that travels between the two patches is an integer random variable extracted from a multinomial distribution. As a concrete example let us consider the migration out from Bangui in the $X_{Bangui}$ compartment. The possible destinations are all the other 136 patches of the network with a probability that decline with the Euclidean distance. The random variables are extracted from:

${Pr}^{multin}\left( X_{Bangui},P_{X_{Bangui}\to X_{1}},\ldots,P_{X_{Bangui}\to X_{136}} \right)$ (9)

with migration probabilities:

- $P_{X_{Bangui}\to X_{j}}=\frac{w_{Bangui,j}}{N_{Bangui}}\Delta t$

where: $X_{Bangui}$ is the number of dogs in Bangui in the $X$ compartment at time *t*; $N_{Bangui}$ is the total number of dogs in Bangui at time *t*. After extracting these numbers from the corresponding distributions, we can calculate the change in the $X_{Bangui}$ population.

## Maximum likelihood estimation

We wrote the likelihood function associated to the observed epidemiological situation in Bangui, i.e. persistence of the disease, size distribution of epidemic waves and absence of population depletion due to the infection, the three events considered as independent.

Through a Monte Carlo procedure [15,16], the set of numerical observations allow reconstructing:

1. The probability $P_{1}(O)$ of observing rabies persistence, where $O$ is the Bernoulli variable equal to 1 if rabies persists and 0 otherwise; such probability is defined as the fraction of runs where persistence occurs.
2. The discrete probability $P_{2,i}\{s_{i}\}$ , associated to the size of the epidemic waves in Bangui, defined as the fraction of times that a certain size $\{s_{i}\}$ of an epidemic wave is observed. We indicate with $\left\{ s_{i}^{*} \right\}$ the set of empirically observed wave sizes. Since the phylogenetic analysis conducted in Bangui [6] suggests that the wave like behaviour observed through surveillance is given by the extinction of local chains of transmission coupled with the re-introduction of new lineages from outside, we can consider the waves sizes as statistically independent variables. Therefore we factorize the total probability $P_{2}(\left\{ s_{i} \right\})$ in the product of the distribution for each size of the epidemic wave: $P_{2}\left( \left\{ s_{i} \right\} \right)=\prod_{i} P_{i}(s_{i})$.
3. The discrete probability $P_{3}(\Delta N)$ of observing a given variation in the Central Africa Republic dog population, defined as the fraction of times that a certain difference with the initial population is observed.

The three combined probabilities lead to the likelihood function defined as follows:

$\mathcal{L(}R_{0},$birth rate$|O=1,\left\{ s_{i} \right\}=\left\{ s_{i}^{*} \right\},\Delta N=0) =\mathcal{L}_{1}($ $R_{0},$birth rate$|O=1)\mathcal{L}_{2}(R_{0},$birth rate$|\left\{ s_{i} \right\}=\left\{ s_{i}^{*} \right\})\mathcal{L}_{3}(R_{0},$birth rate$\left| \Delta N=0 \right)=P_{1}(O=1|R_{0},birth rate)P_{2}\left( \left\{ s_{i} \right\}=\left\{ s_{i}^{*} \right\}|R_{0},birth rate \right)P_{3}\{\Delta N=0|R_{0},birth rate\}$

To compute confidence intervals we used the profile log-likelihood method [17].

## Details of the experimental scenarios

In this section we provide the properties considered by each scenario explored in the main paper. S2 Table reports the number of patches involved, the number of links, the number of connected components, the size of the largest connected component and the total canine population. Fig 2 we report the rabies persistence probability in Bangui exploring higher values of $R_{0}$ higher compared to the other scenarios.

**Table 2.** **Scenarios tested**. Properties of each experimental scenario where we test rabies persistence in the domestic dog population.

| Scenario | Number of patches (%) | Number of links (%) | Number of connected components | Number of patches of the largest connected component (%) | Dog population (%) |
| --- | --- | --- | --- | --- | --- |
| Central African Republic | 137 (100%) | 9,316 (100%) | 1 | 137 (100%) | 76,992 (100%) |
| only Bangui | 1 (0.7%) | 0 | 1 | 1 (100%) | 36,089 (47%) |
| Bangui neighbourhood | 14 (10%) | 91 (1%) | 123 | 14 (100%) | 38,129 (49%) |
| no Bangui | 136 (99%) | 9,180 (99%) | 2 | 135 (99%) | 40,903 (53%) |
| no Bangui neighbourhood | 123 (89%) | 7,503 (80%) | 15 | 122 (99%) | 38,863 (50%) |
| only urban patches | 58 (42%) | 1,653 (18%) | 79 | 58 (100%) | 69,250 (90%) |
| only rural patches | 79 (58%) | 3,081 (33%) | 58 | 79 (100%) | 7,742 (10%) |
| only short travels | 137 (100%) | 183 (2%) | 55 | 26 (19%) | 76,992 (100%) |
| only medium travels | 137 (100%) | 951 (10%) | 10 | 127 (93%) | 76,992 (100%) |
| only long travels | 137 (100%) | 8,182 (88%) | 1 | 137 (100%) | 76,992 (100%) |
| no long travels | 137 (100%) | 1,134 (12%) | 6 | 127 (93%) | 76,992 (100%) |
| no medium travels | 137 (100%) | 8,365 (90%) | 1 | 137 (100%) | 76,992 (100%) |
| no short travels | 137 (100%) | 9,133 (98%) | 1 | 137 (100%) | 76,992 (100%) |

**Fig 2. Dog rabies persistence probability in Bangui.** Persistence probability of rabies in the domestic dog population of Bangui as a function of the basic reproductive number $R_{0}$ and of the annual birth rate. Results are obtained using empirically distributed incubation and infectious periods. Predicted persistence probability is equal to zero. We use the same color scale as in the main text for the sake of comparison.

## Sensitivity analysis

Here we provide the results of the sensitivity analysis on various aspects of the model.

6.1 Carrying capacity

We made a sensitivity analysis on the carrying capacity *K* since previous works indicate that this quantity may vary substantially depending on the geographical and ecological context [5,18]. Our sensitivity analysis indicates a rather stable behavior of the predicted persistence probability of rabies in the country, except for the smallest value of $K$, $K=2$ (Fig 3). This value however does not allow the expected oscillatory behaviour of the domestic dog population.

**Fig 3. Impact of the carrying capacity *K*.** Persistence probability as a function of the annual birth rate for the best estimates of $R_{0}$ for the different values of the carrying capacity tested. The two dotted lines indicate 80% and 95% persistence probability. The vertical blue dotted line represents the maximum likelihood estimate for the birth rate.

6.2 Sensitivity of surveillance system and initial conditions

In the main paper, we used the sensitivity of the surveillance system reported in Bourhy et al., i.e. a detection probability ρ = 20%. For sensitivity, we tested in addition other values, i.e. ρ = 5, 10, 50%, to explore the model behavior varying the sensitivity of the surveillance system.

The persistence probability profile is robust for ρ = 5, 10%, as shown in Fig 4.

If sensitivity is higher, i.e. ρ = 50%, slightly larger values of the reproductive number would be needed to achieve similar values of persistence. Such value however indicates a very efficient surveillance system (i.e. 1 out of 2 rabid dogs detected) that is considered not to be realistic for Central African Republic.


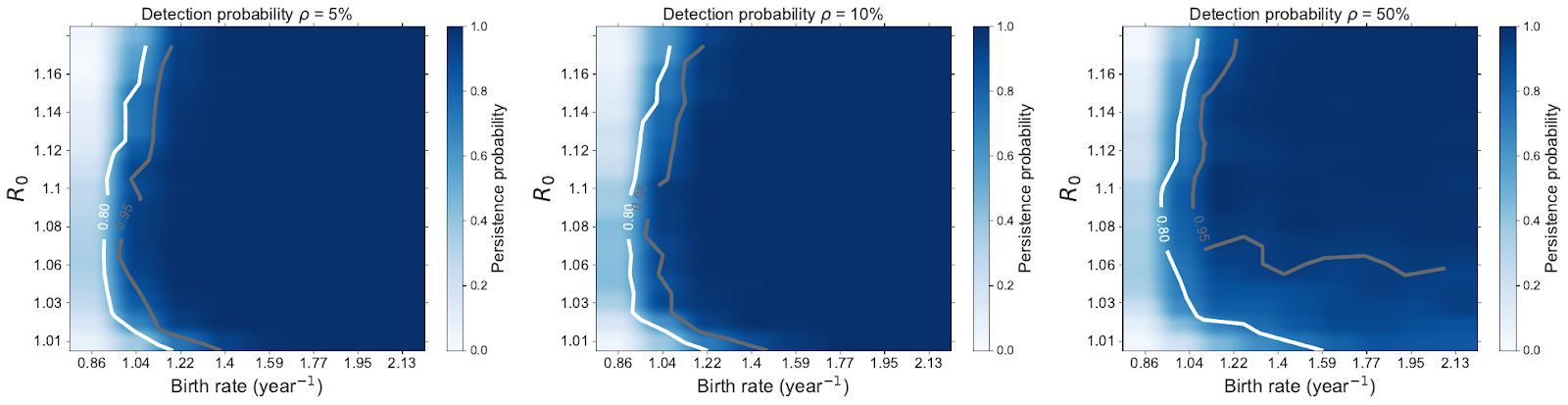


**Fig 4. Impact of sensitivity of surveillance system and associated initial conditions.** Predicted persistence probability of rabies in the domestic dog population of Central African Republic as a function of the basic reproductive number $R_{0}$ and of the annual birth rate. Results are obtained using empirically distributed incubation and infectious periods and testing detection probabilities equals to ρ = 5, 10, 50% (from left to right), to explore the model behavior varying the sensitivity of the surveillance system.

This analysis also corresponds to assess the impact of different initial conditions for the numerical results. Given the endemic situation in the country, and since there is no empirical evidence of a difference in endemic patterns between urban and rural areas, we used Bangui’s surveillance data to estimate the proportion of latent and infectious individuals in all patches of the model. Varying the sensitivity of the surveillance system, as done above, also corresponds to changing the initial conditions. Fig 4 shows that these changes would not alter the model results under realistic conditions.

## Additional numerical results

In this Section we provide additional results complementing those of the main text.

Fig 5 shows the variation of the population dog size over time.

Fig 6 shows the results of the synthetic scenario of considering the full Central African Republic except Bangui, or Bangui with its neighborhood.

**Fig 5. Domestic dog population in Central African Republic over time.** Simulated number of domestic dogs in the Central African Republic. Colors correspond to five different numerical runs, and the red dotted line corresponds to the initial estimated population.


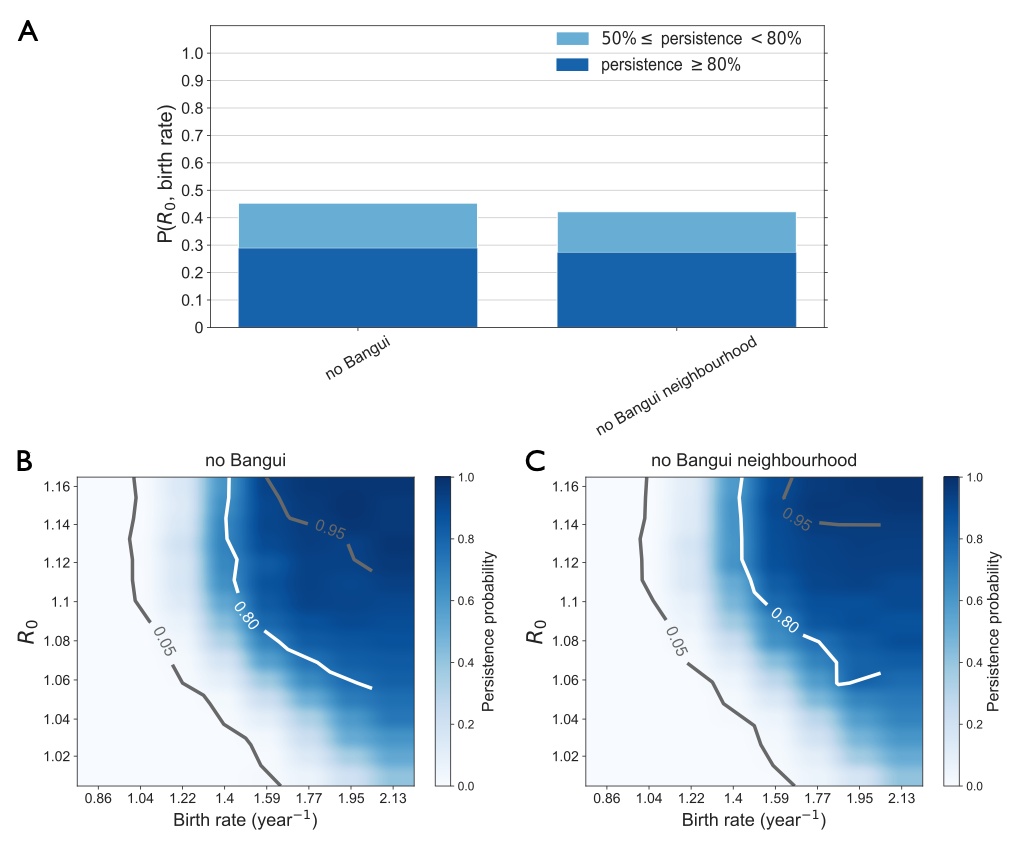


**Fig 6. A** Likelihood to have persistence probability between 50% and 80%, or larger than 80% (light blue/dark blue stacked histogram) in the full parameter space ${(R}_{0},$ birth rate$)$ investigated and for different experimental scenarios (panels B and C). **B, C** Predicted persistence probability of rabies in the domestic dog population as a function of the basic reproductive number $R_{0}$ and of the annual dog birth rate for the scenarios considering: all patches population without Bangui B; all patches population without Bangui and its neighboring patches C.

**References**

1. Wearing HJ, Rohani P, Keeling MJ. Appropriate Models for the Management of Infectious Diseases. Ellner SP, editor. PLoS Med. 2005;2: e174. doi:10.1371/journal.pmed.0020174

2. Feng Z, Xu D, Zhao H. Epidemiological Models with Non-Exponentially Distributed Disease Stages and Applications to Disease Control. Bull Math Biol. 2007;69: 1511–1536. doi:10.1007/s11538-006-9174-9

3. Diekmann O, Heesterbeek JAP, Roberts MG. The construction of next-generation matrices for compartmental epidemic models. J R Soc Interface. 2010;7: 873–885. doi:10.1098/rsif.2009.0386

4. Hampson K, Dushoff J, Cleaveland S, Haydon DT, Kaare M, Packer C, et al. Transmission Dynamics and Prospects for the Elimination of Canine Rabies. Rupprecht CE, editor. PLoS Biol. 2009;7: e1000053. doi:10.1371/journal.pbio.1000053

5. Bilinski AM, Fitzpatrick MC, Rupprecht CE, Paltiel AD, Galvani AP. Optimal frequency of rabies vaccination campaigns in Sub-Saharan Africa. Proc R Soc B Biol Sci. 2016;283: 20161211. doi:10.1098/rspb.2016.1211

6. Bourhy H, Nakouné E, Hall M, Nouvellet P, Lepelletier A, Talbi C, et al. Revealing the Micro-scale Signature of Endemic Zoonotic Disease Transmission in an African Urban Setting. Parrish C, editor. PLOS Pathog. 2016;12: e1005525. doi:10.1371/journal.ppat.1005525

7. Butler JRA, Bingham J. Demography and dog-human relationships of the dog population in Zimbabwean communal lands. Vet Rec. 2000;147: 442–446. doi:10.1136/vr.147.16.442

8. Knobel DL, Cleaveland S, Coleman PG, Fèvre EM, Meltzer MI, Miranda MEG, et al. Re-evaluating the burden of rabies in Africa and Asia. Bull World Health Organ. 2005;83: 360–368.

9. WorldPop. Data. 2014.

10. Linard C, Gilbert M, Snow RW, Noor AM, Tatem AJ. Population Distribution, Settlement Patterns and Accessibility across Africa in 2010. Schumann GJ-P, editor. PLoS ONE. 2012;7: e31743. doi:10.1371/journal.pone.0031743

11. Linard C, Tatem AJ. Large-scale spatial population databases in infectious disease research. Int J Health Geogr. 2012;11: 7. doi:10.1186/1476-072X-11-7

12. Tricou V, Bouscaillou J, Kamba Mebourou E, Koyanongo FD, Nakouné E, Kazanji M. Surveillance of Canine Rabies in the Central African Republic: Impact on Human Health and Molecular Epidemiology. Zinsstag J, editor. PLoS Negl Trop Dis. 2016;10: e0004433. doi:10.1371/journal.pntd.0004433

13. Talbi C, Lemey P, Suchard MA, Abdelatif E, Elharrak M, Jalal N, et al. Phylodynamics and Human-Mediated Dispersal of a Zoonotic Virus. Emerman M, editor. PLoS Pathog. 2010;6: e1001166. doi:10.1371/journal.ppat.1001166

14. Ferguson EA, Hampson K, Cleaveland S, Consunji R, Deray R, Friar J, et al. Heterogeneity in the spread and control of infectious disease: consequences for the elimination of canine rabies. Sci Rep. 2015;5: 18232. doi:10.1038/srep18232

15. Balcan D, Hu H, Goncalves B, Bajardi P, Poletto C, Ramasco JJ, et al. Seasonal transmission potential and activity peaks of the new influenza A (H1N1): a Monte Carlo likelihood analysis based on human mobility. BMC Med. 2009;7: 45.

16. Poletto C, Pelat C, Levy-Bruhl D, Yazdanpanah Y, Boelle P, Colizza V. Assessment of the Middle East respiratory syndrome coronavirus (MERS-CoV) epidemic in the Middle East and risk of international spread using a novel maximum likelihood analysis approach. Eurosurveillance. 2014;19: 3.

17. Venzon DJ, Moolgavkar SH. A Method for Computing Profile-Likelihood-Based Confidence Intervals. Appl Stat. 1988;37: 87. doi:10.2307/2347496

18. Leung T, Davis SA. Rabies Vaccination Targets for Stray Dog Populations. Front Vet Sci. 2017;4.
